# A novel tp53-associated nomogram to predict the overall survival in patients with pancreatic cancer

**DOI:** 10.1186/s12885-021-08066-2

**Published:** 2021-03-31

**Authors:** Xun Liu, Bobo Chen, Jiahui Chen, Shaolong Sun

**Affiliations:** Department of Pancreas and Endocrine Surgery, Shengjing Hospital of China Medical University, No. 36 Sanhao Street, Heping District, Shenyang, 110004 Liaoning China

**Keywords:** TP53 mutation, Survival prediction, WGCNA, Nomogram, Pancreatic Cancer

## Abstract

**Background:**

Gene mutations play critical roles in tumorigenesis and cancer development. Our study aimed to screen survival-related mutations and explore a novel gene signature to predict the overall survival in pancreatic cancer.

**Methods:**

Somatic mutation data from three cohorts were used to identify the common survival-related gene mutation with Kaplan-Meier curves. RNA-sequencing data were used to explore the signature for survival prediction. First, Weighted Gene Co-expression Network Analysis was conducted to identify candidate genes. Then, the ICGC-PACA-CA cohort was applied as the training set and the TCGA-PAAD cohort was used as the external validation set. A TP53-associated signature calculating the risk score of every patient was developed with univariate Cox, least absolute shrinkage and selection operator, and stepwise regression analysis. Kaplan-Meier and receiver operating characteristic curves were plotted to verify the accuracy. The independence of the signature was confirmed by the multivariate Cox regression analysis. Finally, a prognostic nomogram including 359 patients was constructed based on the combined expression data and the risk scores.

**Results:**

TP53 mutation was screened to be the robust and survival-related mutation type, and was associated with immune cell infiltration. Two thousand, four hundred fifty-five genes included in the six modules generated in the WGCNA were screened as candidate survival related TP53-associated genes. A seven-gene signature was constructed: Risk score = (0.1254 × ERRFI1) - (0.1365 × IL6R) - (0.4400 × PPP1R10) - (0.3397 × PTOV1-AS2) + (0.1544 × SCEL) - (0.4412 × SSX2IP) – (0.2231 × TXNL4A). Area Under Curves of 1-, 3-, and 5-year ROC curves were 0.731, 0.808, and 0.873 in the training set and 0.703, 0.677, and 0.737 in the validation set. A prognostic nomogram including 359 patients was constructed and well-calibrated, with the Area Under Curves of 1-, 3-, and 5-year ROC curves as 0.713, 0.753, and 0.823.

**Conclusions:**

The TP53-associated signature exhibited good prognostic efficacy in predicting the overall survival of PC patients.

**Supplementary Information:**

The online version contains supplementary material available at 10.1186/s12885-021-08066-2.

## Introduction

Pancreatic cancer (PC) has been the 11th most common cancer in 2012. Both the incidence and mortality rates of PC have been increasing in developed countries. PC was the 3rd leading cause of cancer-related mortality in the United States in 2017, and will grow to be the 2nd leading cause of cancer-related mortality in 2030 [1, 2]. As the malignancy with the highest mortality, the survival rate of PC has not been increased despite years of investigation [3]. The primary treatment approach is still surgery, though only 20% of patients will survive over 5 years after pancreatectomy [4]. The main reasons include delayed diagnosis at an advanced stage, ineffective treatment, and poor prognosis. The diagnosis of pancreatic cancer has been generally difficult because it depended on the clinical symptoms, which were not indicative during onset and gradual progression over years. There may be only midepigastric pain, weight loss, malaise, nausea, and fatigue. Most patients diagnosed with PC have developed metastases, leading to poor prognostic outcomes [5]. The detection of pancreatic cancer at early and resectable stage has been proved to have beneficial effects on long-term survival.

Numerous studies have tried to explore the risk factors for PC. Smoking, Alcohol consumption, Obesity and Dietary factors have been proved to increase the risk of PC [2]. Except for the clinical symptoms, biomarkers in blood or biospy have been also developed for PC screen and monitor. Serum cancer antigen 19–9 (CA 19–9) is the only approved marker for clinical management of PC patients [6]. With the advances of proteome and genomes, some specific expression profiles have been revealed in PC patients [7]. These expression profiles may assist in interpreting the hereditary incidence, unpredictable efficacy of clinical treatment, as well as the poor outcomes. A recent study has summarized the selected protein biomarkers in tissue, serum, plasma, and pancreatic juice. The combination of CA 19–9 and other emerging biomarkers improved clinical management of PC [7]. The genetic alterations have also been investigated in PC patients. The study proposed several known frequently mutated genes (including KRAS and TP53) and revealed mutations in critical signaling pathways [4]. Further, a recent meta-analysis involving 9040 patients and 12,496 controls reported five new susceptibility loci for PC [8]. The emerging genetic alterations assisted in the better characterization of the complex diseases.

PC is a heterogeneous disease with various subtypes [9]. More understanding may help in improving the disease management [10]. Here, from another point of view, we explored the gene alterations in tumorigenesis and cancer development of PC based on comprehensive bioinformatic analysis. Overall survival-related mutations were screened and a novel gene signature was developed to predict the overall survival for patients with PC. It may be beneficial to improve the prognosis prediction and post-surgery management.

## Materials and methods

### Data sources

A total of three cohorts were included in this study. Somatic mutation data (*n* = 124) and RNA-sequencing data (*n* = 178) with corresponding clinical data of the TCGA-PAAD cohort were downloaded from the Cancer Genome Atlas (https://cancergenome.nih.gov/). Somatic mutation data of the ICGC-PACA-CA cohort (*n* = 263) and the ICGC-PACA-AU cohort (*n* = 373) with corresponding clinical data were downloaded from the International Cancer Genome Consortium (ICGC) database (https://www.icgc.org). RNA-sequencing data of the ICGC-PACA-CA cohort (*n* = 182) with corresponding clinical data were also downloaded from the International Cancer Genome Consortium (ICGC) database (https://www.icgc.org).

### Data processing and normalization

Somatic mutation data of the TCGA-PAAD cohort were based on VarScan2 [11]. Somatic mutation data of the ICGC-PACA-AU cohort were based on qsnp [12]. Somatic mutation data of the ICGC-PACA-CA cohort were based on MuTect [13]. Synonymous variant data which could not cause the change of amino acid sequence were filtered out. The RNA-sequencing data of the ICGC-PACA-CA and the TCGA-PAAD cohorts were normalized data (FPKM). All expression values were log2-transformed. The batch effect was eliminated with the SVA R package in R 3.6.1. The somatic mutation data of the three cohorts were used to identify the common survival-related gene mutation. The RNA-sequencing data of the ICGC-PACA-CA and the TCGA-PAAD cohorts were used to explore and validate the multi-gene signature to predict the overall survival in patients with pancreatic cancer.

### Identification of survival-related mutations

Somatic mutation data of the three cohorts were extracted and sorted with Perl 5.32.0 (https://www.perl.org/). Mutational frequencies were calculated by the counting method. The top 30 genes with the highest mutation frequency were acquired from the three cohorts separately. Waterfall plots of the mutational landscape were generated with the GenVisR R package [14]. The common mutations were selected and drawn by the Venn diagram. In order to identify robust mutations which were associated with the overall survival, Kaplan-Meier (KM) curves comparing the mutated group with the wild group were plotted by the Survival R package in three cohorts separately. *P* < 0.05 were considered to be indicative of significance. Finally, only TP53 mutation was indicated to be significantly related to the overall survival in all the three cohorts.

### Association between TP53 mutation and tumor mutation burden (TMB)

TMB is an important genetic factor in mediating antitumor immunity. In this study, number of non-synonymous single nucleotide polymorphism (SNP) of each sample was calculated with Perl 5.32.0. TMB scores were estimated as number of variants/the length of exons (38 million). Wilcoxon tests were conducted to compare the differences of TMB between the TP53 mutated and wild groups in the three cohorts.

### Gene set enrichment analysis (GSEA)

To reveal functional differences between PC patients with and without TP53 mutations, GSEA was done in GSEA 4.0.1 software with the gene set c2 (cp.kegg.v.6.2.symbols.gmt). A total of 65 patients without TP53 mutations and 77 patients with TP53 mutations in the TCGA-PAAD cohort were included. The normalized RNA-sequencing data (FPKM) were used in the GSEA. The threshold was set at FDR (false discovery rate) < 0.05 and NES (normalized enrichment score) > 1.5. The results were drawn with the ggplot2 R package.

### Analysis of total leucocyte infiltration and 22 immune cell types’ infiltration pattens

To explore the differences in tumor immune infiltrating cells between TP53-mutated and TP53-wild patients, the estimations of total leucocyte infiltration were performed by the ESTIMATE R package [15], and the fractions of 22 immune cell types (B cells naïve, B cells memory, Plasma cells, T cells CD8, T cells CD4 naïve, T cells CD4 memory resting, T cells CD4 memory activated, T cells follicular helper, T cells regulatory (Tregs), T cells gamma delta, NK cells resting, NK cells activated, Monocytes, Macrophages M0, Macrophages M1, Macrophages M2, Dendritic cells resting, Dendritic cells activated, Mast cells resting, Mast cells activated, Eosinophils and Neutrophils) were calculated with CIBERSORT R script v1.03 [16]. Sixty-five patients without TP53 mutations and 77 patients with TP53 mutations in the TCGA-PAAD cohort were included. Immune scores generated from the ESTIMATE algorithm were used to reflect and compare the total leucocyte infiltrations. The CIBERSORT analysis was conducted by using the default signature matrix at 1000 permutations. Results with *P* ≥ 0.05 were excluded. 35 wild and 60 mutated patients were included for the further analysis. Differentially analysis was performed with the Wilcoxon test and plotted by the vioplot R package. *P* < 0.05 were considered to be statistically significant. Pearson’ correlations among the infiltrations of the 22 immune cell types and the immune scores were plotted with the corrplot R package in the wild and mutated groups separately.

### Screening of survival related TP53-associated genes

To screen candidate genes which were both associated with TP53 mutations and overall survival, WGCNA was applied with the WGCNA R package [17]. There were 67 wild and 82 mutated patients in the TCGA-PAAD cohort with complete overall survival data included. First, outlier samples were excluded by sample clustering. The sample dendrogram and the clinical-traits heatmap were plotted. Then, the scale-free network was constructed with the appropriate soft-threshold power (β) value. The scale-free topology was plotted to show the constructed scale-free network.

The co-expression modules generated from the scale-free network were further plotted with dynamic tree cutting. Modules were merged if their similarity was greater than 0.75 according to dendrogram height. The Pearson’s correlation coefficients between each module and clinical traits were further calculated and plotted. Modules with correlations with TP53 mutation greater than 0.2 (*P* < 0.05) and significantly associated with overall survival (*P* < 0.05) were considered as survival-related TP53-associated modules. The genes included in the modules were screened as candidate survival related TP53-associated genes.

### Developing and validation of TP53-associated prognostic signature

In this study, the gene expression data of the ICGC-PACA-AU cohort were mainly based on microarray platform. While, the gene expression data of the ICGC-PACA-CA and the TCGA-PAAD cohorts were all based on RNA- sequencing platforms. The sample size of the ICGC-PACA-CA cohort was larger than the TCGA-PAAD cohort. So, the ICGC-PACA-CA cohort was applied as the training set and the TCGA-PAAD cohort was used as the external validation set. First, the univariate Cox regression analysis was done to further identify the survival related TP53-associated genes in the training set with the cutoff of *P* < 0.05. The LASSO and stepwise regression analyses were applied to construct the best-fit TP53-related prognostic signature, which could estimate the risk score of every patient. Based on the median risk score of the training set, all patients were divided into high- and low-risk groups in both sets. Kaplan-Meier (KM) curves were used to perform the survival analysis which could compare the overall survival of different groups. The predictive performances at different endpoints (1, 2, 3, 4, and 5 years) were assessed with the receiver operating characteristic (ROC) curves in both cohorts. The alteration trends of risk score, survival time, survival status, and expression levels of the genes included in the signature were further plotted with the pheatmap R package in both cohorts separately.

### Independent prognostic prediction analysis

To explore the independence of the TP53-related signature, univariate and multivariate Cox regression analyses were conducted with the Survival R package. One hundred forty-three patients with complete clinical information of the age, gender, stage, and risk score were included in the ICGC-PACA-CA cohort. One hundred and seventy-five patients with complete clinical information of the age, gender, grade, stage, alcohol, family history, and risk score were included in the TCGA-PAAD cohort. The hazard ratio (HR) and *P* values were plotted. *P* < 0.05 were considered statistically significant. 5-year Receiver Operating characteristic Curves (ROC) of the risk score and other clinical features were plotted with the survivalROC R package. Area Under Curves (AUCs) were applied to compare the accuracies of different factors’ prediction abilities.

### Construction of the prognostic Nomogram

Based on the multivariate Cox regression analysis in the independent prognostic prediction analysis, the risk score was the only robust factor significantly (*P* < 0.05) associated with overall survival in both cohorts. The expression data and the risk scores of the two cohorts were combined. A total of 359 patients were included and the prognostic nomogram was constructed with the rms R package. The survival analysis was carried out by KM plotter. The calibration curve was further plotted using the calibrate function. ROC curves of 1, 3, and 5 years were plotted with the survivalROC R package. Then, we conducted the performance comparison of the signature in our study (referred to as TP53Sig) with eight recently published signatures: 6-mRNA signature from Hou’s study (referred to as HouSig) [18], 4-mRNA signature from Meng’s study (referred to as MengSig) [19], 8-mRNA signature from Meng’s study (referred to as MengSig) [20], 5-mRNA signature from Wu’s study (referred to as WuSig) [21], 7-mRNA signature from Wu’s study (referred to as WuSig) [22], 10-mRNA signature from Yue’s study (referred to as YueSig) [23], 2-mRNA signature from Zhou’s study (referred to as ZhouSig) [24] and 6-LncRNA signature from Deng’s study (referred to as DengSig) [25]. ROC curves of 3 years were plotted and AUC values were calculated using the survivalROC R package.

### Survival analysis in subgroups

To evaluate the predictive level of TP53-related prognostic score in different subgroups (Age>65, Age ≤ 65, Male, Female, Stage I, Stage II, Stage III-IV, Grade 1, Grade 2 and Grade 3–4), Kaplan-Meier (KM) curves were plotted with the Survival R package.

### Relevance with clinical features

The relationships between the risk score and the other clinical features (Age, Gender, Stage and Grade) were explored by using the ggpubr R package in the combined cohort with Kruskal-Wallis test. *P* < 0.05 indicated statistically significant.

### Immune cell infiltration analysis and GSEA

To explore the differences in tumor immune infiltrating cells between the high-risk patients (*n* = 183) and the low-risk patients (*n* = 176), the estimations of total leucocyte infiltration were performed by the ESTIMATE R package and the fractions of 22 immune cell types were estimated with CIBERSORT in R 3.6.1. Immune scores generated from the ESTIMATE algorithm were used to reflect and compare the total leucocyte infiltrations with Wilcoxon test. The CIBERSORT analysis was conducted by using the default signature matrix at 1000 permutations. Results with *P* ≥ 0.05 were excluded. Then, according to the previous Charoentong’s study [26], we further explored the differential expression analysis of 96 immunotherapy-related genes between the 183 high-risk and 176 low-risk patients with Wilcoxon Test. Finally, the functional differences between the high-risk and low-risk groups were explored with gene set enrichment analysis in GSEA 4.0.1 software.

### Statistical analysis

The Kaplan–Meier method was used to perform survival analysis, and the log-rank test was used to assess the difference. Differentially analysis of TMB values, immune scores and 22 immune cell types’ infiltrations were conducted with the Wilcoxon test. Univariate and multivariate Cox regression analysis were used to assess the independence of the risk score. The performances of the signatures were evaluated by the ROC curves. The relationships between the risk score and the other clinical features were explored with Kruskal-Wallis test. *P* < 0.05 indicated statistically significant.

## Results

### Screening TP53 mutation to be robust and survival-related mutation type

**Fig. 1:**
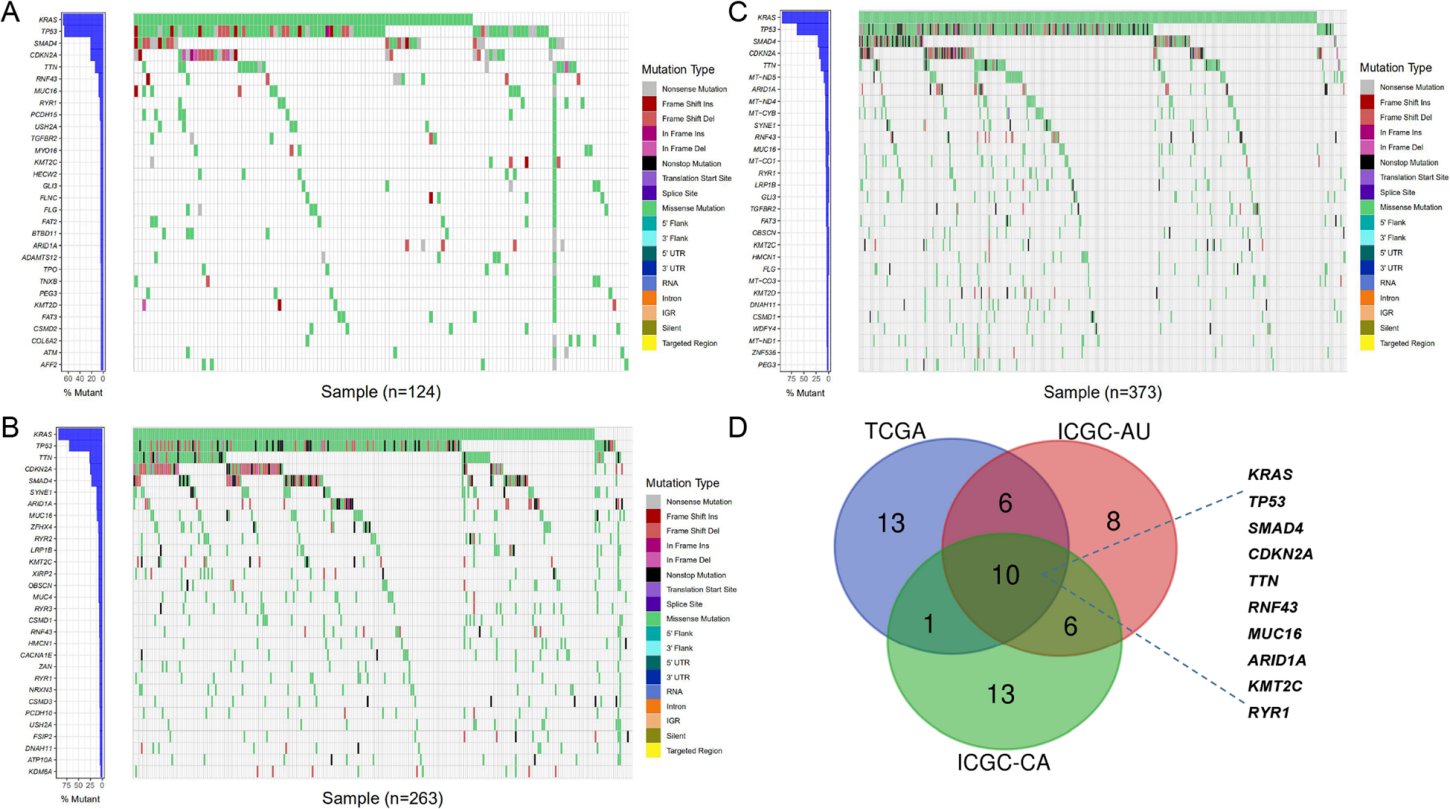
The mutational landscapes of the top 30 genes with the highest mutation frequency. **a** TCGA-PAAD. **b** ICGC-PACA-CA. **c** ICGC-PACA-AU. **d** Venn diagram

**Fig. 2:**
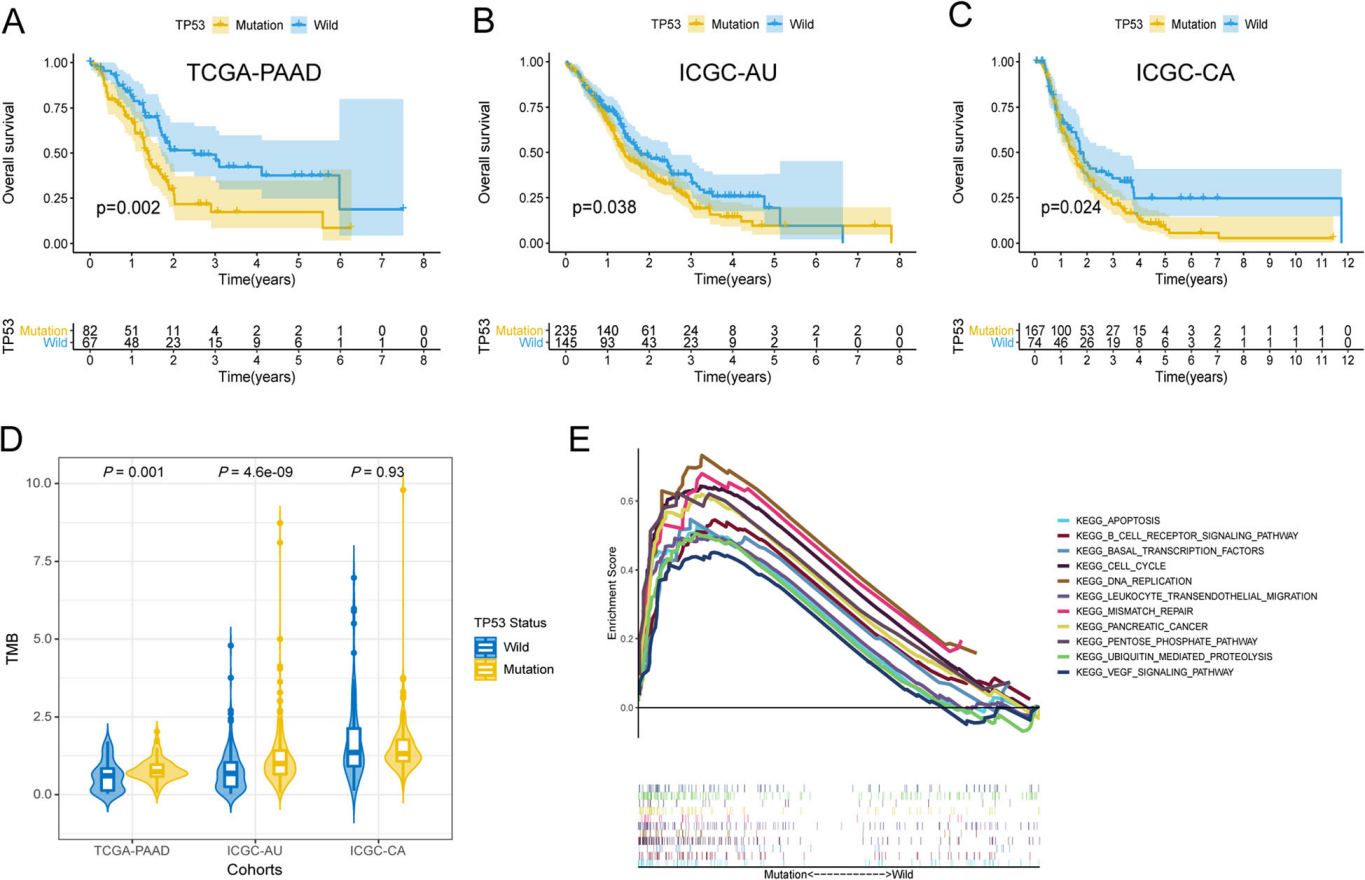
TP53 mutation’s associations with overall survival, TMB and GSEA. **a** Kaplan-Meier curve of TCGA-PAAD cohort. **b** Kaplan-Meier curve of ICGC-PACA-AU cohort. **c** Kaplan-Meier curve of ICGC-PACA-CA cohort. **d** Tumor mutation burden (TMB) analysis**. e** Differences in biological functions between TP53-mutated and TP53-Wild patients by GSEA

Mutational landscapes of the top 30 genes with the highest mutation frequency in the three cohorts were plotted separately (Fig. 1a-c). As shown in the results, KRAS and TP53 mutations were the top two mutation types. The mutation frequencies of SMAD4, CDKN2A, and TTN mutations ranked the third to the fifth in the three cohorts. The common ten (KRAS, TP53, SMMAD4, CDKN2A, TTN, RNF43, MUC16, ARID1A, KMT2C, and RYR1) mutations were drawn by the Venn diagram (Fig. 1d). In order to explore whether these mutations were associated with the overall survival robustly, Kaplan-Meier (KM) curves comparing the mutated group with the wild group were performed in the three cohorts separately. Finally, TP53 mutation was indicated to be the only robust and survival-related mutation type. Kaplan-Meier (KM) curves were shown (Fig. 2a-c). TP53-mutated patients had significantly worse overall survival in the three cohorts (*P* < 0.05).

### Association between TP53 mutation and tumor mutation burden (TMB)

TMB values of TP53-mutated group were significantly higher in TCGA-PAAD cohort (*P* = 0.001) and ICGC-PACA-AU cohort (*P* < 0.001). However, no significant difference was found in the ICGC-PACA-CA cohort (*P* = 0.93) (Fig. 2d). Whether TP53 mutation affects TMB needs to be further explored.

### TP53 mutation was associated with immune cell infiltration

**Fig. 3:**
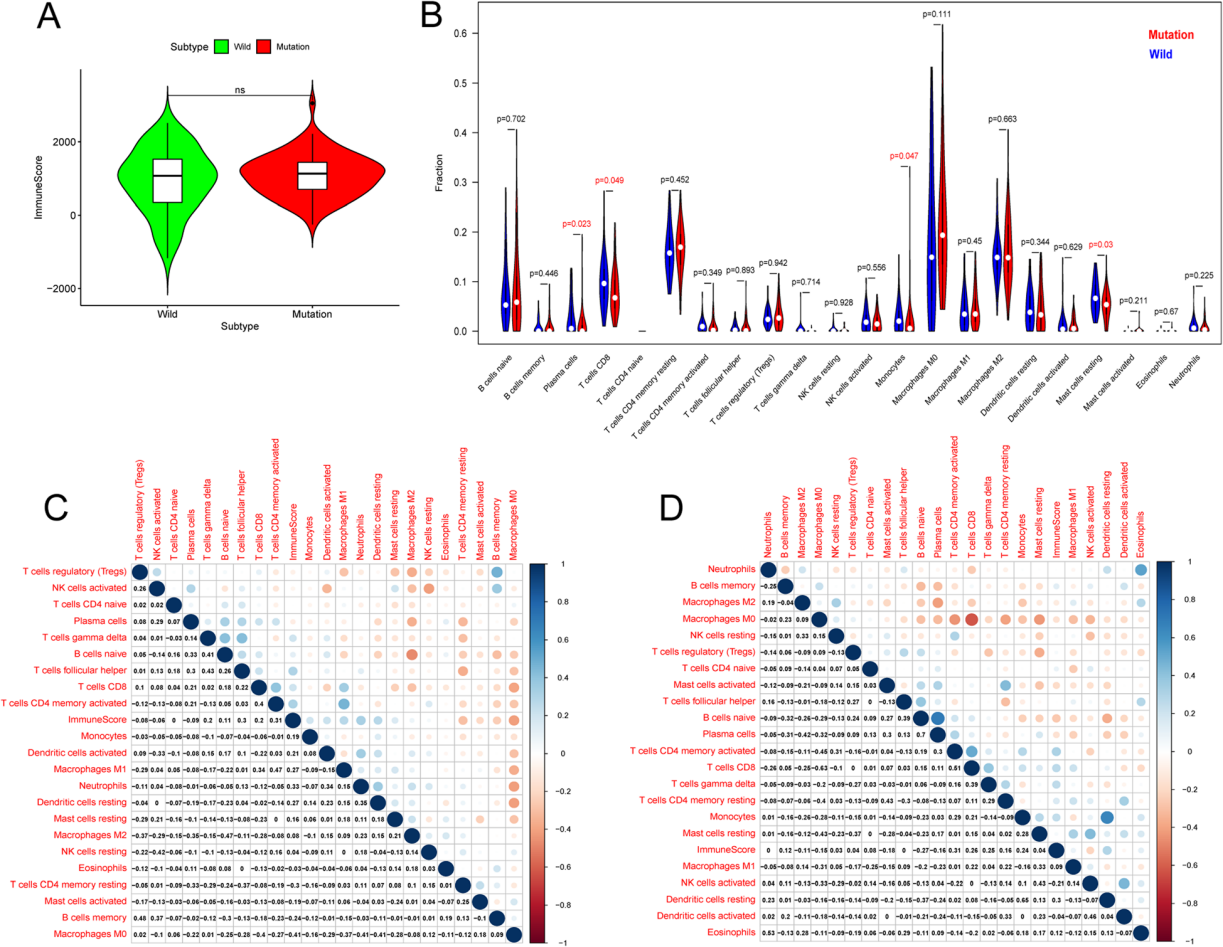
TP53 mutation was associated with immune cell infiltration. **a** Comparison of the immune scores**. b** Differences of 22 immune cell types’ infiltrations between TP53-mutated and TP53-Wild patients. **c** The Pearson’ correlations among the 22 immune cell types’ infiltrations and the immune scores in TP53-Wild patients. **d** The Pearson’ correlations among the 22 immune cell types’ infiltrations and the immune scores in TP53- mutated patients. *P* < 0.05 indicated statistically significant. Note: ns: not significant

GSEA results indicated that the B cell receptor signaling pathway and leukocyte transendothelial migration were significantly enriched in the TP53-mutated patients (Fig. 2e). Immune scores reflecting the total leucocyte infiltrations by ESTIMATE showed no significant difference between the two groups **(**Fig. 3a**).** However, fraction pattens of the 22 immune cell types by CIBERSORT were different. In details, the fractions of plasma cells (*P* = 0.023), T cells CD8 (*P* = 0.049), Monocytes (*P* = 0.047) and Mast cells resting (*P* = 0.03) in TP53-mutated patients were significantly lower than those in TP53-wild patients (Fig. 3b). Moreover, the immune score had the most positive correlation with the fraction of Neutrophils (R = 0.33) and the most negative correlation with the fraction of Macrophages M0 (R = − 0.41) in the wild group (Fig. 3c). And the immune score had the most positive correlation with the fraction of T cells CD4 memory activated (R = 0.31) and the most negative correlation with the fraction of B cells naïve (R = − 0.27) in the mutated group (Fig. 3d).

### Screening of candidate TP53-associated genes by WGCNA

**Fig. 4:**
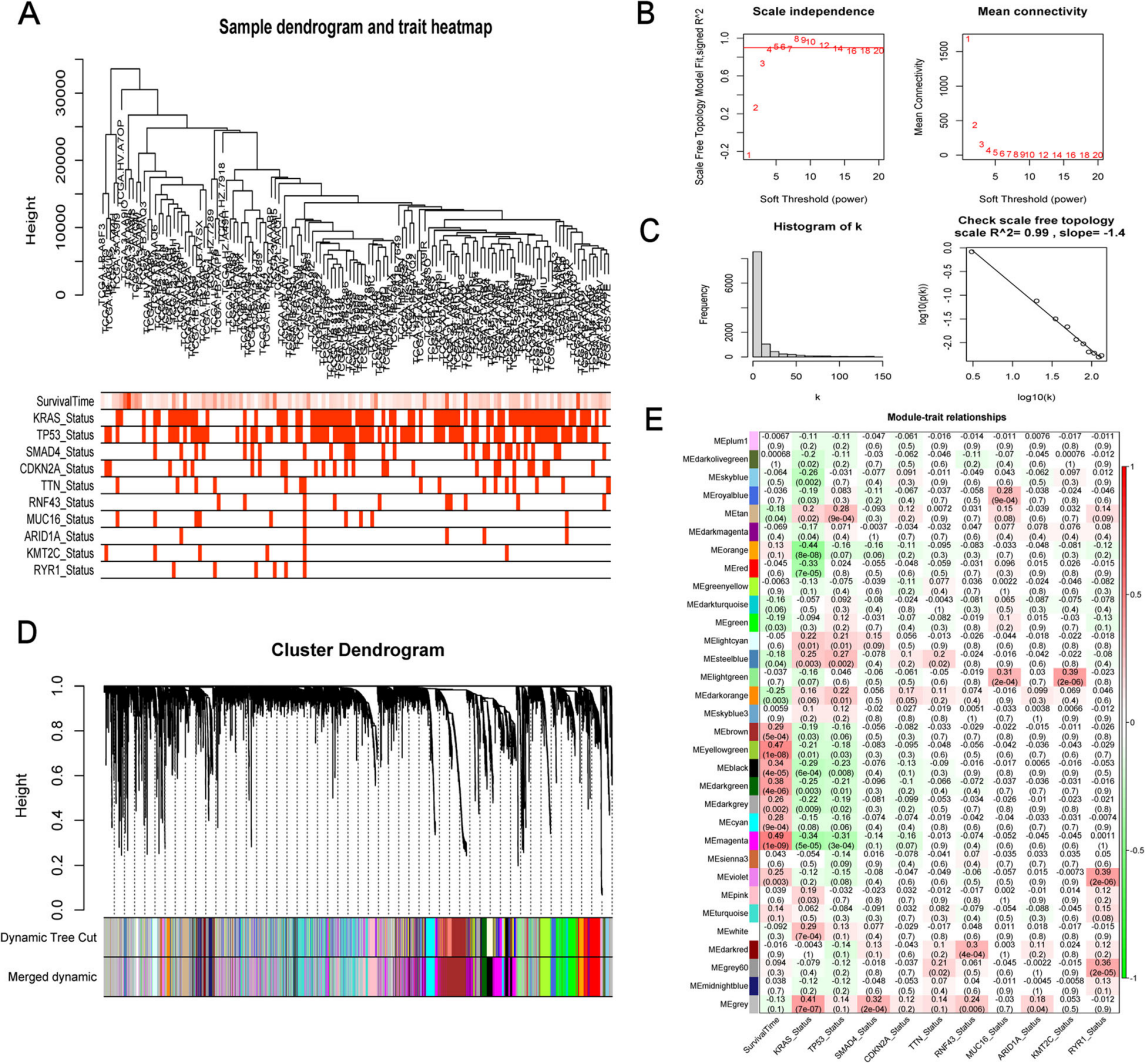
Screening of candidate TP53-associated genes by WGCNA. **a** Sample dendrogram and clinical-traits heatmap. **b** Construction of the scale-free network with the soft-threshold power (β) value = 8. **c** Scale-free topology plot with R^2^ = 0.99 and slope = − 1.4. **d** Co-expression modules plotted with dynamic tree cutting. **e** The Pearson’s correlation coefficients between each module and clinical traits. *P* < 0.05 indicated statistically significant

To screen candidate TP53-associated genes, WGCNA was performed in the TCGA-PAAD cohort. First, six outlier samples were excluded by sample clustering. The sample dendrogram and the clinical-traits heatmap was plotted (Fig. 4a). Then, the scale-free network was constructed with the soft-threshold power (β) value as 8 (Fig. 4b). The constructed scale-free network was shown by the scale-free topology, with R^2^ = 0.99 and slope = − 1.4 (Fig. 4c). The co-expression modules generated from the scale-free network were further plotted with dynamic tree cutting (Fig. 4d). The Pearson’s correlation coefficients between each module and clinical traits (Additional file 1) were plotted (Fig. 4e). Six Modules (magenta, darkgreen, black, darkorange, steelblue, tan) with correlations with TP53 mutation greater than 0.2 (*P* < 0.05) and significantly related with the overall survival (*P* < 0.05) were considered as survival related TP53-associated modules. A total of 2455 genes included in the six modules were screened as candidate survival related TP53-associated genes.

### Developing and validation of TP53-related prognostic signature

**Fig. 5:**
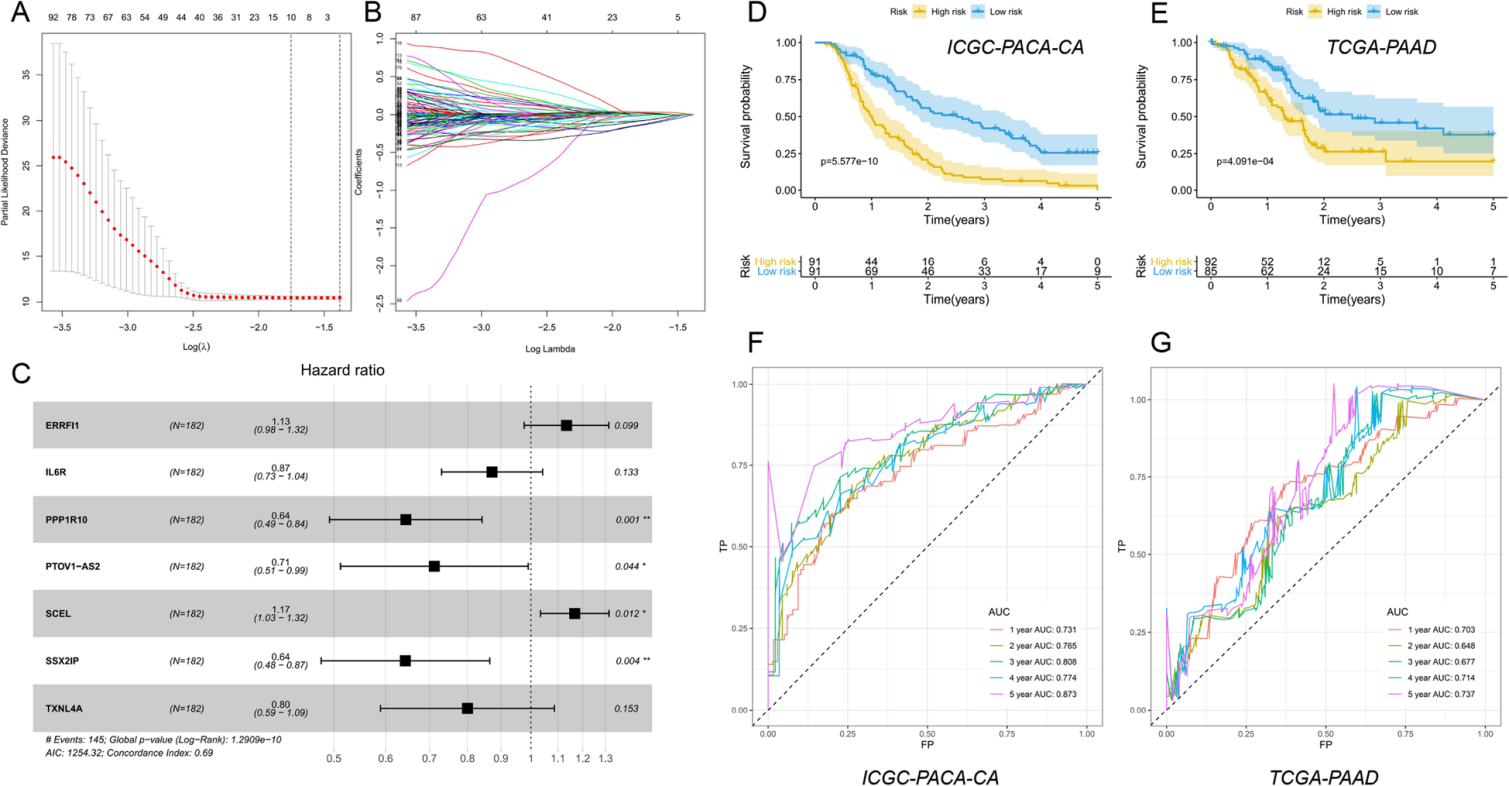
Developing and validation of TP53-associated prognostic signature. **a** Tuning parameter (lambda) in the LASSO regression analysis. **b** Dynamic coefficient profiling of the LASSO regression analysis. **c** The hazard ratios (HRs) in the stepwise regression analysis. **d** Kaplan-Meier curve of the training cohort. **e** Kaplan-Meier curve of the external validation cohort. **f** The 1-, 2-, 3-, 4-, and 5-year ROC curves of the training cohort. **g** The 1-, 2-, 3-, 4-, and 5-year ROC curves of the external validation cohort. *P* < 0.05 indicated statistically significant

**Fig. 6:**
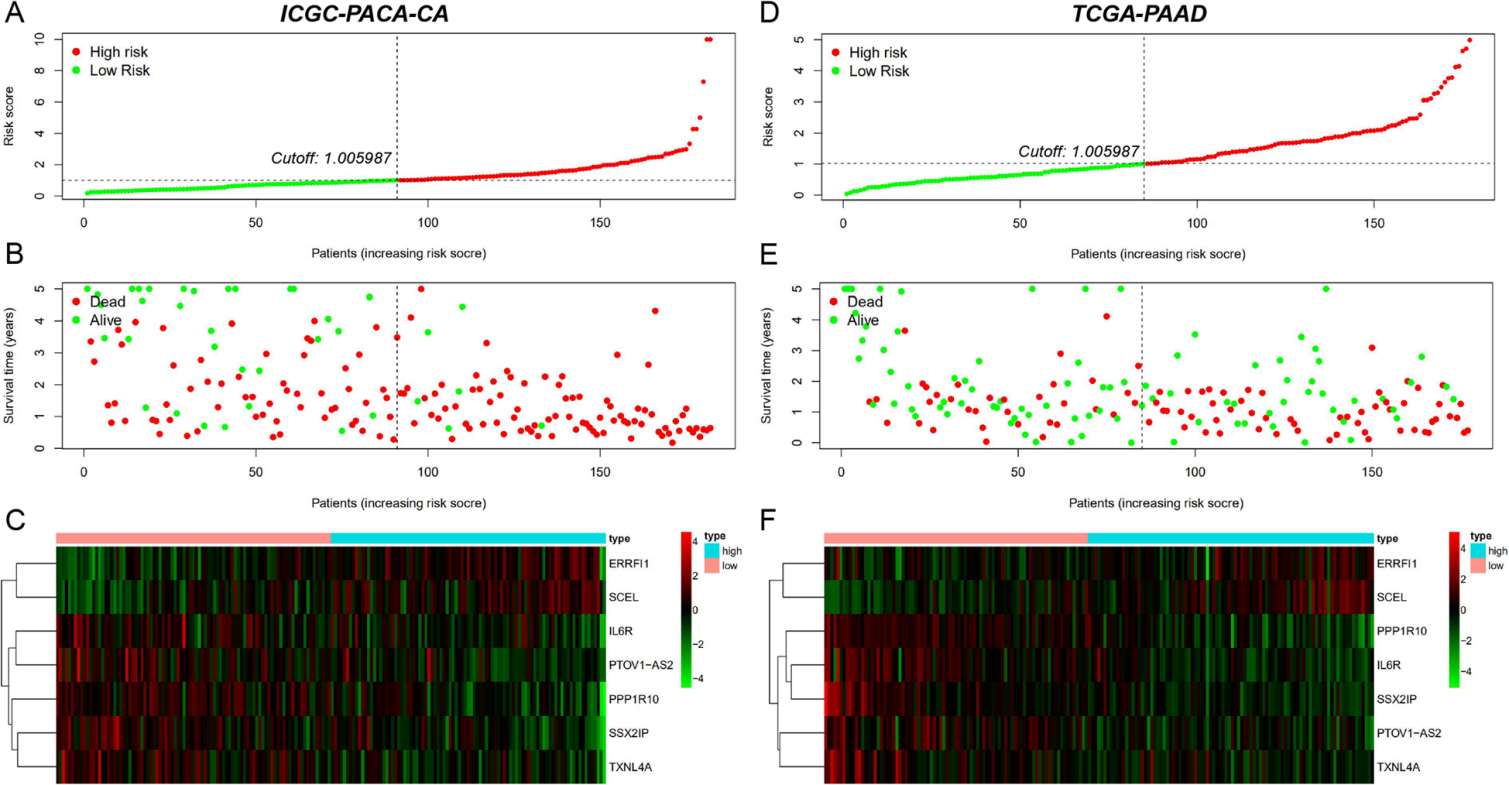
Alteration trends of risk score, survival and expression levels of the genes included in the signature. **a**, **d** The distribution of calculated risk scores. **b**, **e** The survival time and status of high- and low- risk patients. **c**, **f** Expression heatmap of the seven genes included in the signature

The ICGC-PACA-CA cohort (*n* = 182) was applied as the training set and the TCGA-PAAD cohort (*n* = 177) was used as the external validation set. Patient characteristics in the training and the testing cohorts were shown (Table 1). First, univariate cox regression analysis was done in the training set and a total of 316 genes were identified with *P* < 0.05. LASSO regression analysis was further applied and 10 genes were screened (Fig. 5a, b). Then, the stepwise regression analysis was used and constructed the best-fit TP53-related prognostic signature calculating the risk score of each patient. Seven genes were included in the signature calculating the risk score with the formula: risk score = (0.1254 × ERRFI1) - (0.1365 × IL6R) - (0.4400 × PPP1R10) - (0.3397 × PTOV1-AS2) + (0.1544 × SCEL) - (0.4412 × SSX2IP) – (0.2231 × TXNL4A). The hazard ratios (HRs) were plotted (Fig. 5c). Based on the median risk score 1.005987 of the training set, all patients were divided into high- and low-risk groups in both training and external validation sets. Kaplan-Meier (KM) curves showed the overall survival of the high-risk patients was worse than that of the low-risk patients (*P* < 0.001) (Fig. 5d-e). The ROC curves of 1-, 2-, 3-, 4-, and 5-year were plotted, with the AUC of 0.731, 0.765, 0.808, 0.774, and 0.873 in the training set (Fig. 5f) and the AUC of 0.703, 0.648, 0.677, 0.714, and 0.737 in the external validation set (Fig. 5g). Alteration trends of risk score, survival time, survival status and the included genes’ expression levels were further plotted separately (Fig. 6a-f).

**Table 1:** Patient characteristics in the training and the testing cohorts

ICGC-PACA-CA cohort (Training)				TCGA-PAAD cohort (Testing)			
Characteristics		Number of cases	% (percentage)	Characteristics		Number of cases	% (percentage)
Age				Age			
	<=65	78	42.86		<=65	93	52.54
	> 65	86	47.25		> 65	84	47.46
	Unknown	18	9.89		Unknown	0	0
Gender				Gender			
	Male	99	54.40		Male	97	54.80
	Female	82	45.05		Female	80	45.20
	Unknown	1	0.55		Unknown	0	0
Stage				Stage			
	Stage I	51	28.02		Stage I	22	12.43
	Stage II	85	46.70		Stage II	148	83.62
	Stage III-IV	8	4.40		Stage III-IV	7	3.95
	Unknown	38	20.88		Unknown	0	0
Grade				Grade			
	G1	NA	NA		G1	31	17.51
	G2	NA	NA		G2	94	53.11
	G3–4	NA	NA		G3–4	50	28.25
	Unknown	NA	NA		Unknown	2	1.13

Note: *NA* Not Available

### Independence of the TP53-related prognostic signature

**Fig. 7:**
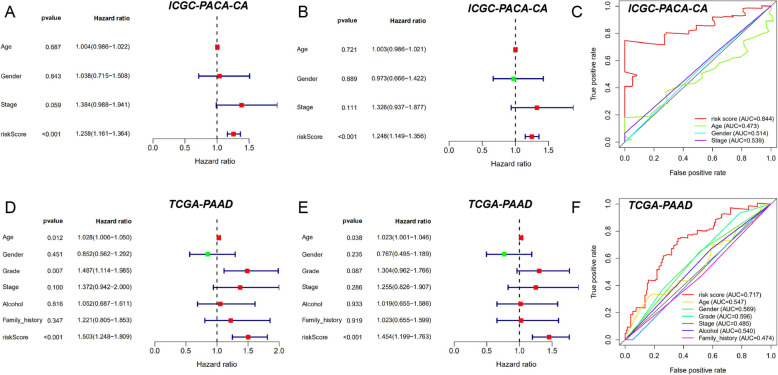
Independence of the TP53-related prognostic signature. **a** Univariate Cox regression results of ICGC-PACA-CA. **b** Multivariate Cox regression results of ICGC-PACA-CA. **c** The 5-year ROC curve of clinical features in ICGC-PACA-CA. **e** Univariate Cox regression results of TCGA-PAAD. **f** Multivariate Cox regression results of TCGA-PAAD. **g** The 5-year ROC curve of clinical features in TCGA-PAAD. *P* < 0.05 indicated statistically significant

Univariate and multivariate Cox regression analyses were performed to explore the independence of the TP53-related prognostic signature from other clinical features. The hazard ratio (HR) and *P* values were plotted. The 5-year Receiver Operating Characteristic Curves (ROC) of the risk score and other clinical features were plotted. The risk score was proved to be the only variable significantly associated with overall survival in the univariate (*P* < 0.001) and multivariate (*P* < 0.001) Cox regression results in the ICGC-PACA-CA cohort (Fig. 7a, b). The 5-year ROC curve was plotted with the highest value of 0.844 than that of the other clinical features included (Fig. 7c). The risk score (*P* < 0.001), age (*P* = 0.012), and grade (*P* = 0.007) were the three variables significantly associated with overall survival in the univariate analysis of the TCGA-PAAD cohort (Fig. 7d). The risk score (*P* < 0.001) and age (*P* = 0.038) were the two significant variables in the multivariate analysis of the TCGA-PAAD cohort (Fig. 7e). The 5-year ROC curve was also plotted with the highest value of 0.717 than that of the other clinical features included (Fig. 7f). These results indicated that the risk score based on the TP53-related prognostic signature was independent from other clinical features.

### Construction of the prognostic Nomogram

**Fig. 8:**
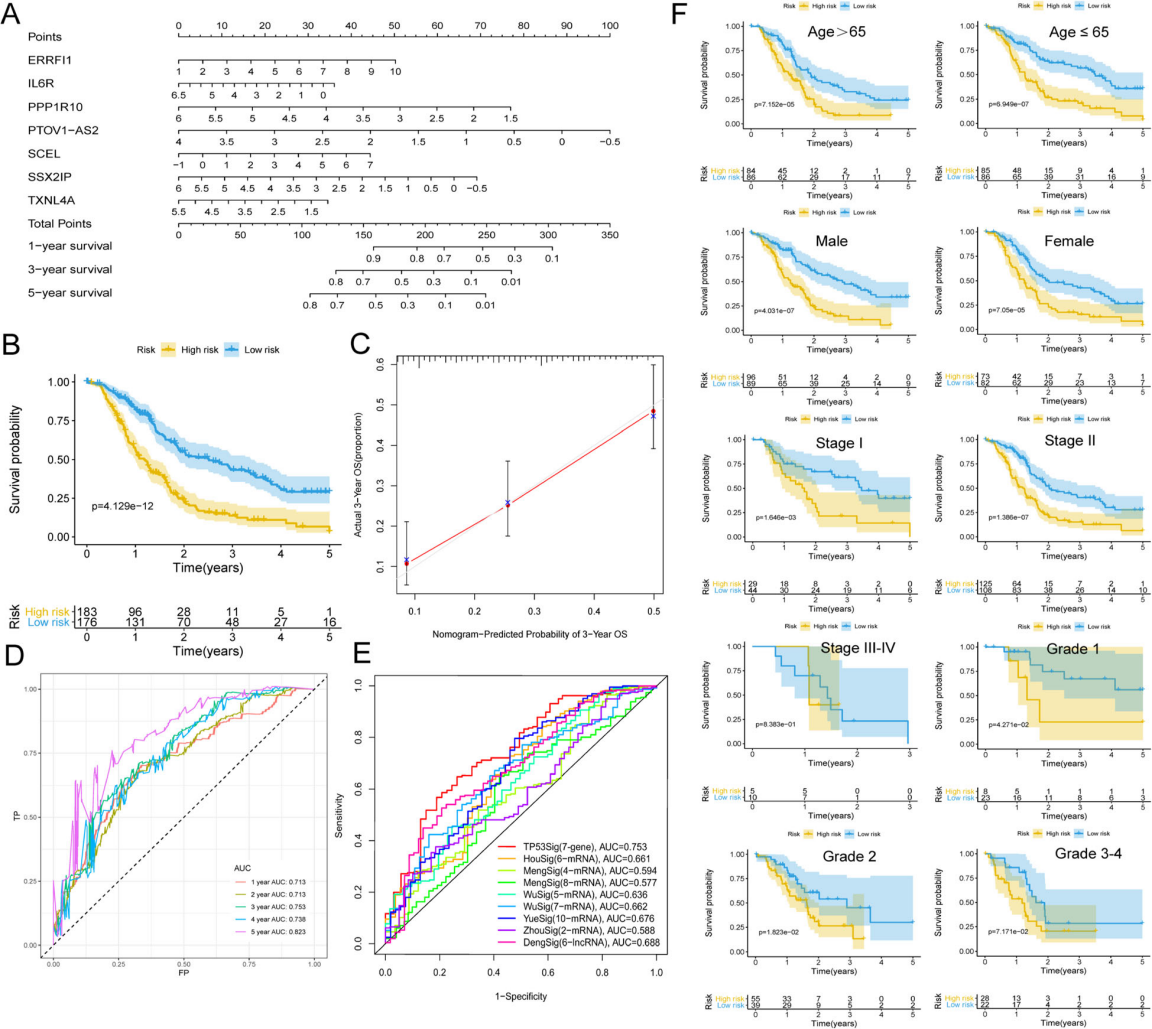
Construction of the prognostic nomogram and the prediction accuracy. **a** The prognostic nomogram including 359 patients. **b** KM curve of the nomogram in the combined cohort. **c** The calibration curve of 3 years. **d** The 1-, 3-, and 5-year ROC curves. **e** The ROC analysis at 3 years of overall survival for the signature in our study (referred to as TP53Sig) and other published signatures. **f** Kaplan-Meier (KM) curves in subgroups. *P* < 0.05 indicated statistically significant

The risk score was the only robust factor significantly (*P* < 0.05) associated with overall survival in both cohorts. The expression data and the risk scores of the two cohorts were combined. Three hundred and fifty-nine patients were included and the prognostic nomogram was conducted (Fig. 8a). The survival analysis was carried out by KM plotter (Fig. 8b). The calibration curve of 3 years was well calibrated (Fig. 8c). The ROC curves of 1-, 3-, and 5-year were plotted, with the AUC of 0.713, 0.753, and 0.823 (Fig. 8d).

ROC curves of 3 years indicated that the signature in our study had the highest AUC value **(**Fig. 8e).

Kaplan-Meier (KM) curves in subgroups also showed good performance except in Stage III-IV and G3–4 groups (Fig. 8f).

### Relevance with clinical features

**Fig. 9:**
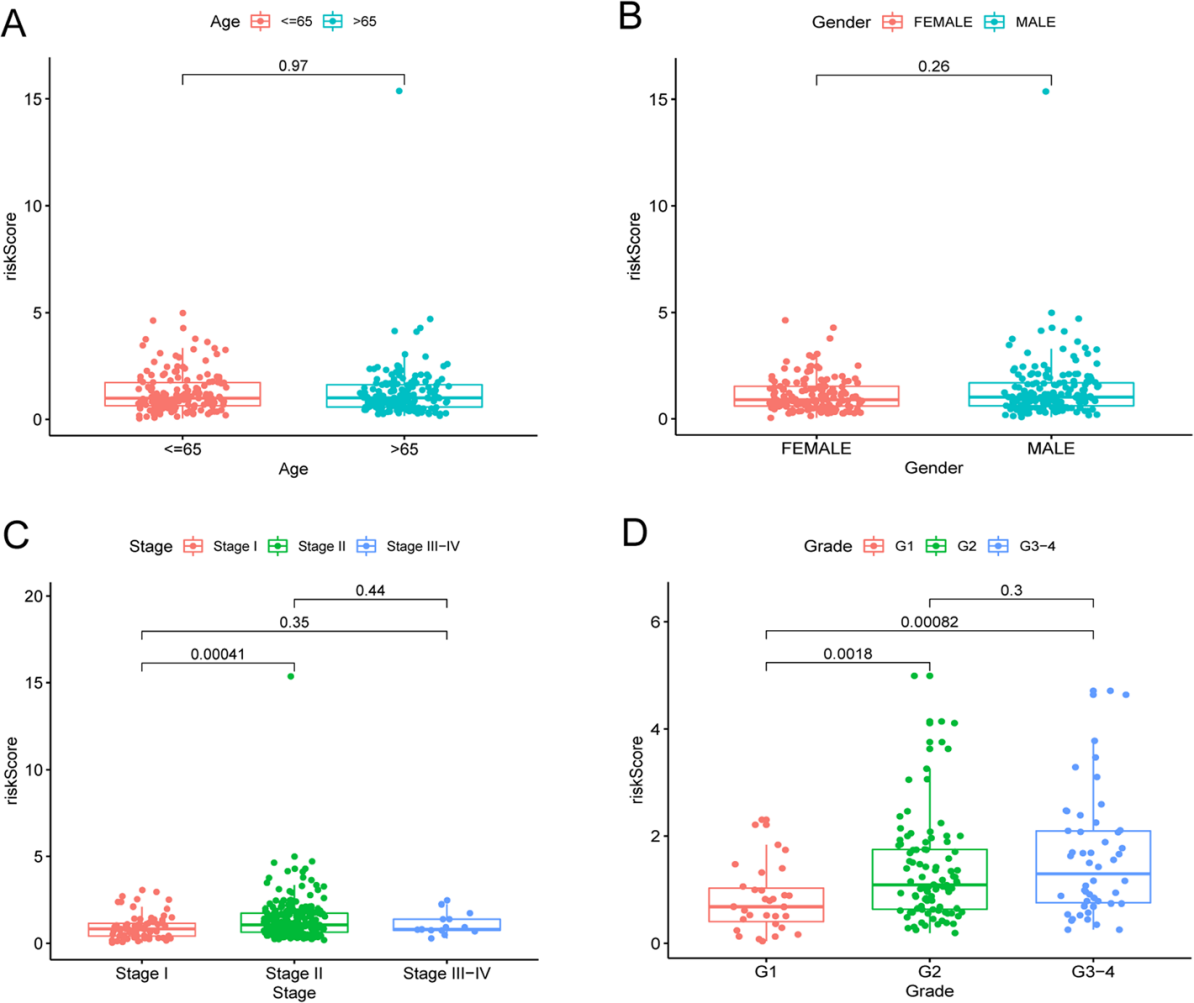
The relationships between risk score and clinical features. **a** Relationships between risk score and the Age, Gender, Stages in the combined cohort. **b** Relationships between risk score and the Grade in the TCGA-PAAD cohort. *P* < 0.05 indicated statistically significant. Note: the data of Grade were not provided in the ICGC-PACA-CA dataset

There were no significant relationships between the risk score and Age or Gender in the combined cohort (Fig. 9a, b). Patients in Stage I had significantly lower risk scores than patients in Stage II (*P* < 0.001) (Fig. 9c). Patients in G1 had significantly lower risk scores than patients in G2 and G3–4 (*P* < 0.01) (Fig. 9d). No significant differences were revealed between Stage I and Stage III-IV, Stage II and Stage III-IV and G2 and G3–4 groups (Fig. 9c, d). The sample size of Stage III-IV (*n* = 15) was small, which might affect the statistical accuracy. In general, the higher the Stage or Grade is, the higher the risk score will be.

### Immune cell infiltration analysis and GSEA

**Fig. 10:**
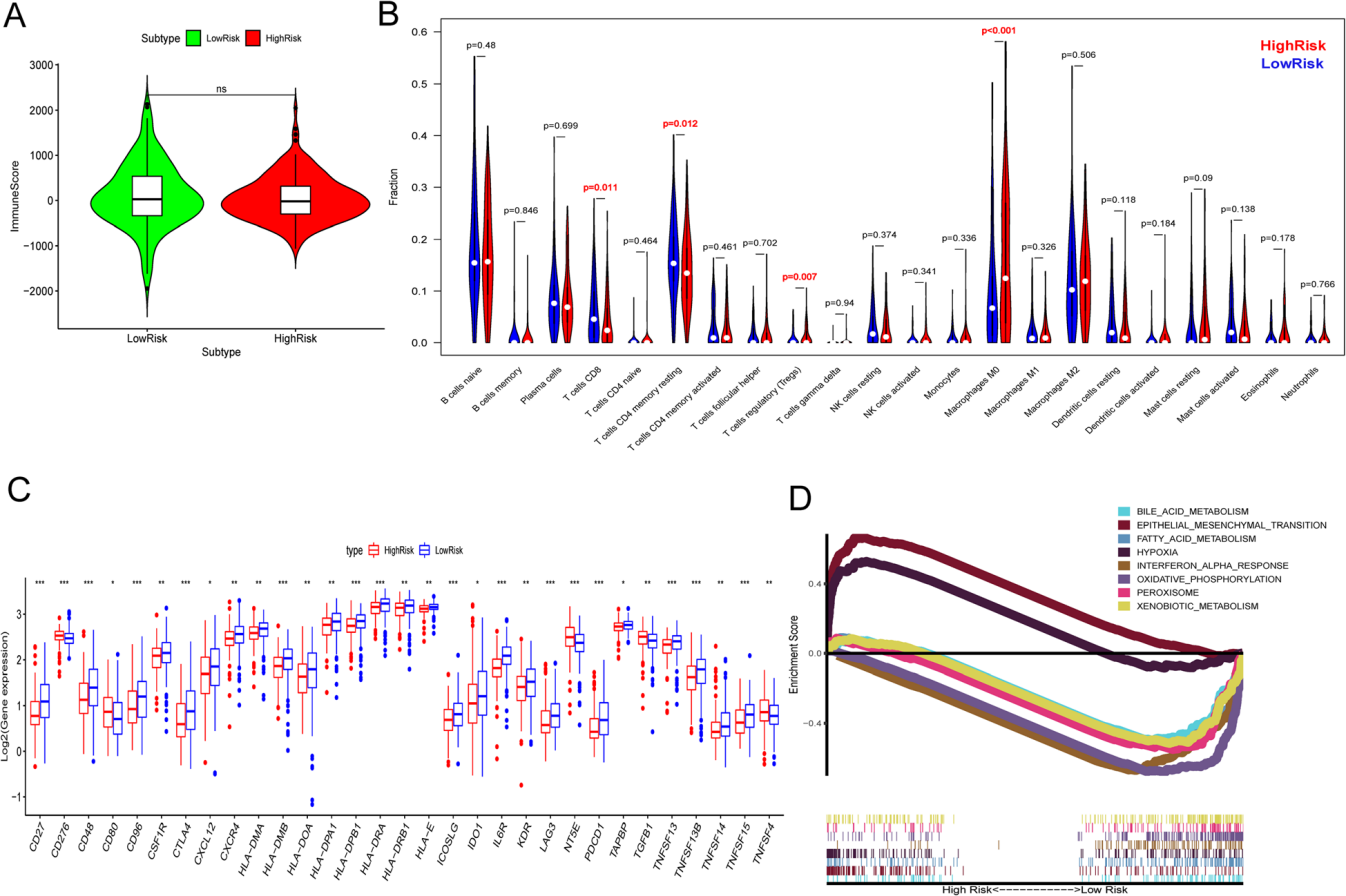
Immune cell infiltration analysis and GSEA in high- and low-risk groups. **a** Comparison of the immune scores**. b** The fractions of 22 immune cell types estimated with CIBERSORT and the differences between high- and low- risk patients. **c** Differential expression analysis of immunotherapy-related genes**. d** Differences in biological functions by GSEA. Note: *** *P* < 0.001, ** *P* < 0.01, * *P* < 0.05

Immune scores by ESTIMATE showed no significant difference between the high-risk and low-risk groups (Fig. 10a). However, fractions of T cells CD8 (*P* = 0.011), T cells CD4 memory resting (*P* = 0.012), and T cells regulatory (Tregs) (*P* = 0.007) in high-risk group were significantly lower (Fig. 10b). The fraction of Macrophages M0 in high-risk group was significantly higher (Fig. 10b). We further explored the differential expression analysis of 96 immunotherapy-related genes **(**Additional file 2**)**. A total of 31 genes were identified, of which 26 genes’ expression levels were lower in the high-risk group. This might indicate that the immune activity in the high-risk group was weaker than that in the low-risk group (Fig. 10c)**.** GSEA results indicated that Epithelial-Mesenchymal Transition and Hypoxia were the most significantly enriched functions in the high-risk patients (Fig. 10d).

## Discussion

Gene alterations have been determined in 97% of patients with PDAC, including point mutations, amplifications, deletions, translocations, and inversions [27]. *TP53* encoded tumor suppressor TP53, which transcriptionally activated target genes for resisting cellular stresses, then inducing growth arrest or apoptosis [28]. TP53 mutation has been observed in 50–75% of PDAC cases, which would initiate activating mutation of the KRAS gene. Mutant TP53 promoted lymph node metastasis and escaped from KrasG12D-induced growth arrest/senescence in PDAC [29]. In clinical practices, the detection of mutant and expression profile of TP53 and associated genes may improve the diagnosis and screen for PC [30, 31]. The sample can be the pancreatic Juice, less-invasive serum exosomes or even non-invasive stool [32, 33]. However, until now, although TP53 have been closely associated with PC, the roles of TP53 have not been elucidated. More information on its upstream and downstream genes would be required [27]. Our bioinformatic analysis has also proved the roles of TP53 in PDAC. Firstly, KRAS and TP53 mutations were observed as the top two genes with the highest mutation frequency. Secondly, TP53 mutation was indicated to be the only robust and survival-related mutation type. Thirdly, TP53-mutated patients showed significantly worse overall survival than TP53-wild patients in included cohorts.

Based on above results, more genes associated with TP53 mutations were further identified. Key modules including 2455 genes were preliminarily screened with WGCNA and Pearson’s correlation. The key 316 genes were secondly screened with univariate Cox regression analysis. Then, 10 most critical genes were identified with LASSO. Finally, the best-fit TP53-related prognostic signature involved 7 genes were constructed with stepwise regression analysis, as well as corresponding coefficients. In the formula for calculating risk score, the high levels of ERRFI1, SCEL increased risk of poor prognosis, while the high levels of IL6R, PPP1R10, PTOV1-AS2, SSX2IP, TXNL4A suggested decreasing risk. The identified 7 genes were potential candidates of biomarkers for prognosis prediction of PDAC [34].

In previous studies, *ERBB receptor feedback inhibitor 1* (*ERRFI1*) was reported as an important regulatory gene. It regulated AKT/EGFR signaling in an EGFR-dependent manner. In EGFR-low cells, ERRFI1 activated AKT and promoted proliferation and chemotherapy resistance. In EGFR-high cells, reduced ERRFI1 led to active EGFR and increased cell proliferation [35]. As a key gene targeted AKT/EGFR signaling, ERRFI1 may be a binding target for some miRNAs and lncRNAs in various cancers, such as cholangiocarcinoma [36, 37]. Until now, the effects of ERRFI1 on PDAC have not been revealed, and its effects in other cancers suggested that it may be promising therapeutic target and biomarker. Interleukin-6 receptor (IL-6R) was the receptor of IL-6. The blocking agents that combined with IL-6 and IL-6R may be potential anti-inflammatory drugs, and some of them may be anti-cancer agents [38]. As an important effector in several signaling pathways, IL6R was also proposed as a new therapeutic target for some cancers [39, 40]. For example, IL-6R participated IL-6R /STAT3/miR-204 feedback loop contributed to chemo-resistance of epithelial ovarian cancer cells [41]. IL-6R participated several important pathways in tumor development and chemo-resistance. The suppression of IL-6R may function in these pathways thus affecting the prognosis. The same to ERRFI1, the roles of IL-6R in PDAC have not been revealed, which worth further investigation. *SCEL* encoded Sciellin (SCEL), which was a precursor of the cornified envelope firstly identified in mammalian keratinizing tissue [42]. SCEL was a mesenchymal-to-epithelial transition inducer dynamically regulated during the metastasis. Thus it may be a site for regulating the colorectal cancer hepatic metastasis [43]. *SSX2IP* encoded Synovial Sarcoma X breakpoint 2 Interacting Protein (SSX2IP) has been revealed to play various roles in human cancers. SSX2IP was known as leukaemia associated antigen [44, 45]. SSX2IP promoted the tumorgenesis and progression of hepatocellular carcinoma and contributed to the drug resistance, enabling it a new biomarker and specific target in hepatocellular carcinoma [46]. High levels of SSX2IP were associated with aggressive pathological features and poor outcomes in nasopharyngeal carcinoma [47]. Few studies have been performed on PPP1R10, PTOV1 antisense 2 (PTOV1-AS2) and Thioredoxin-like protein 4A(TXNL4A). Considering the malignancy of PDAC, relatively less studies could be retrieved, both on the biomarkers or therapeutic targets. The bioinformatic analysis has been a useful tool for exploring promising candidates for further investigation.

## Conclusions

With the screened gene candidates, the TP53-associated signature exhibited good prognostic efficacy in predicting the overall survival of PC patients. In the training and validation cohorts, the 1-, 3-, and 5-year ROC curves were plotted according to the risk score, presenting relatively high AUC. The prognostic nomogram including 359 patients was also constructed to calculate the risk score, which would facilitate the further clinical applications. There are also some limitations in our study. Firstly, the gene expression and somatic cell mutations data were obtained from cancerous tissues. However, the results should be further verified in other types of samples, such as serum and stool. As the less-invasive and non-invasive specimens will tend to promote the early screen and detection of PDAC. Secondly, the association between TP53 mutation and tumor mutation burden should be verified in larger cohort. Thirdly, whether the nomogram could predict immunotherapy or chemotherapy needs further explorations in the future.

## Supplementary Information

**Additional file 1.**

**Additional file 2.**

## Data Availability

The data used and analyzed in this study are available from the corresponding author on reasonable request.

## Abbreviations

PC: Pancreatic cancer
KM: Kaplan-Meier
TMB: Tumor mutation burden
GSEA: Gene set enrichment analysis
WGCNA: Weighted Gene Co-expression Network Analysis
LASSO: Least absolute shrinkage and selection operator
ROC: Receiver operating characteristic
AUCs: Area Under Curves
TCGA: The Cancer Genome Atlas
ICGC: International Cancer Genome Consortium
FDR: False discovery rate
NES: Normalized enrichment score
HR: Hazard ratio
